# Generating exosome subtypes: diverse membrane origins and mergers

**DOI:** 10.1016/j.tcb.2026.03.009

**Published:** 2026-10

**Authors:** Deborah C.I. Goberdhan, Adrian L. Harris, Clive Wilson

**Affiliations:** 1 Nuffield Department of Women’s and Reproductive Health, University of Oxford, Oxford, UK; 2 Department of Oncology, University of Oxford, Oxford, UK; 3 Department of Physiology, Anatomy and Genetics, University of Oxford, Oxford, UK

**Keywords:** extracellular vesicle, intraluminal vesicle, multivesicular endosome, recycling endosome, late endosome, regulated secretion

## Abstract

Exosomes are formally defined as extracellular vesicles, which are formed in compartments with endosomal origin by the inward budding of the endosomal limiting membrane. Recent analyses of the dynamic events within exosome-generating compartments have overturned the dogma that only late endosomal membranes produce exosomes. It is now clear that recycling endosomal, autophagic, regulated secretory, and other organelle membranes contribute to exosome production. In this opinion article, we discuss studies demonstrating the critical roles of membrane origins and mergers, together with intracompartmental microenvironments, in generating intraluminal vesicle and exosome subtypes with diverse physiological and pathological functions, both inside and outside the secreting cell. These findings provide significant opportunities to develop novel strategies that overcome the current challenges of detecting and targeting disease-relevant exosomes.

## Highlights

Exosome-generating multivesicular endosomes include not only late endosomes but also recycling endosomes and fusion compartments with nonendosomal contributions.
Nonendosomal membranes, including those from autophagosomes, mitochondria, and regulated secretory compartments, contribute to the limiting membrane of multivesicular endosomes and intraluminal vesicles, expanding intraluminal vesicle diversity and exosome functions.
Exosome subtypes are further diversified by specialised mechanisms controlling their production and cargo-specific loading, many of which are enhanced by disease-relevant cellular stresses.
Intraluminal vesicles have intracellular roles prior to secretion, for example, in compartment maturation events and physiological and pathological protein aggregation.
Exosome subtype characterisation will facilitate and optimise the use of extracellular vesicles in diagnosis, therapeutic targeting, and biodelivery.

## Exosomes and other extracellular vesicles

Communication between cells is mediated not only by secreted signalling molecules but also by **extracellular vesicles (EVs)** (see Glossary), complex lipid bilayer-bound structures that carry a diverse range of proteins, nucleic acids, and other bioactive molecules [1]. EVs are implicated in diverse aspects of physiological and pathological signalling [2]. Thought to be secreted by all cells, they are broadly categorised into three classes: plasma membrane-derived **ectosomes**, whose diverse functional activities are reviewed elsewhere 3, 4; **exosomes**, initially generated as **intraluminal vesicles (ILVs)** within **endosomes** termed **multivesicular endosomes (MVEs)**, or potentially lysosomes via the inward budding of the limiting membrane; and nonendosomal ‘exosomes’, derived from other nonendosomal **multivesicular bodies (MVBs)**, most notably **nuclear envelope-derived MVBs (NE-MVBs)** 5, 6, 7 (Figure 1A). These definitions will be used throughout this review. Although some plasma membrane EVs, such as large oncosomes and apoptotic bodies, can be separated by size [1], it remains challenging to separate heterogeneous mixtures of exosomes and ectosomes secreted by cells, which are referred to as **small EVs (sEVs)** [15].

**Figure 1:**
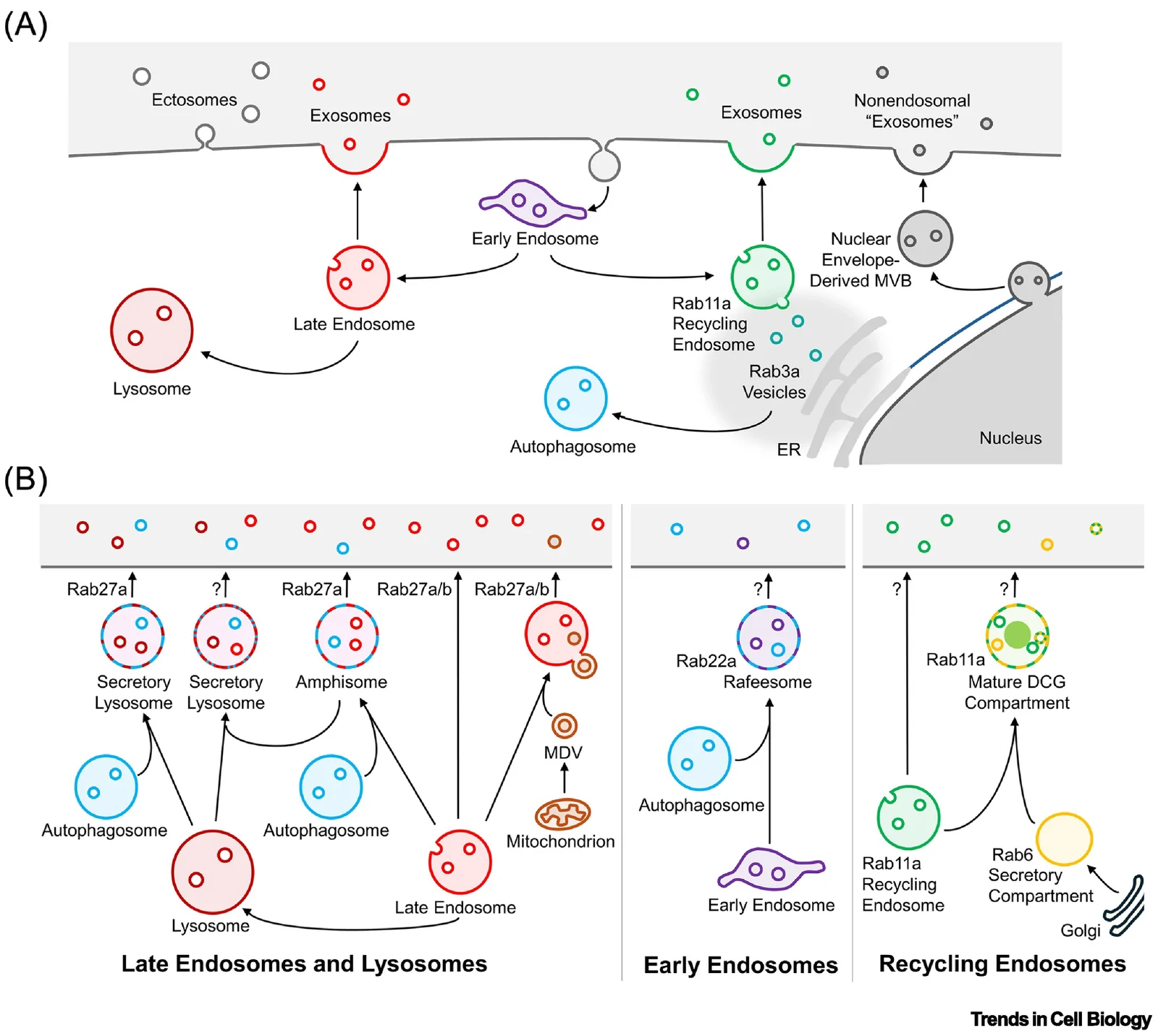
Small extracellular vesicle and exosome identity is impacted by membrane origins and mergers. (A) Small extracellular vesicles (sEVs) can be generated by the outward budding of the plasma membrane to form ectosomes, direct fusion with the plasma membrane of late and recycling multivesicular endosome (MVE) membranes, leading to the secretion of exosomes [1], and fusion of nonendosomal multivesicular bodies (MVBs), producing nonendosomal 'exosomes' [5]. Each of these different sEVs has been reported to have diverse signalling activities. Rab3a-positive vesicles can also bud from Rab11a-positive recycling endosome limiting membranes at an endoplasmic reticulum (ER) contact site. These vesicles then merge to form the enveloping double membrane of autophagosomes [8], which, as shown in (B), can contribute to endosome-derived membranes and resulting exosomes. (B) Nonendosomal inputs increase the diversity of intraluminal vesicle (ILV) membranes and secreted exosomes. Autophagosomes can fuse with late endosomal MVEs to form amphisomes [9]. Amphisomes can themselves merge with lysosomes to form specialised secretory lysosomes [10]. In addition, autophagosomes can directly fuse with lysosomes to form alternative secretory lysosomes [11] or with early endosomes to form Rab22a-labelled rafeesomes [12]. It has also been reported that mitochondrion-derived vesicles (MDVs) can transfer ILVs produced from the inner mitochondrial membrane to MVEs [13]. Recycling endosome fusion with Golgi-derived regulated secretory compartments triggers formation of an aggregated protein dense core and can form ILVs with mixed membrane and protein cargos derived from both contributing compartments [14]. Such mixing has yet to be clearly shown for other endosome/nonendosome fusion compartments. DCG: dense-core granule.

Here, we focus on recent studies demonstrating that endosome-derived exosomes are themselves heterogeneous, with their structure and functions determined by diverse compartmental origins and combinations of membranes. Compartment fusion can introduce nonendosome-derived ILVs and subsequently affect the composition of exosome-forming membrane invaginations. These differences influence exosome biogenesis regulatory mechanisms and can substantially alter protein, nucleic acid, and lipid cargos. The upshot is that only a small proportion of secreted sEVs may be responsible for specific physiological and pathological signalling functions.

## Diverse endosomal and nonendosomal membranes contribute to exosome heterogeneity

The assumption that exosome-generating MVEs are exclusively associated with late endosomal trafficking was challenged by analysis using an *in vivo* model of exosome biogenesis based on *Drosophila* prostate-like secondary cells, which have highly enlarged endosomal and secretory compartments (Figure 2), combined with complementary human cell line studies. This work led to the identification of an exosome subtype made in Rab11a-labelled **recycling endosomes (REs)**, termed **Rab11a-exosomes** (Figures 1A and 2), which has specific physiological and pathological functions 16, 17, 19, 20, 21.

**Figure 2:**
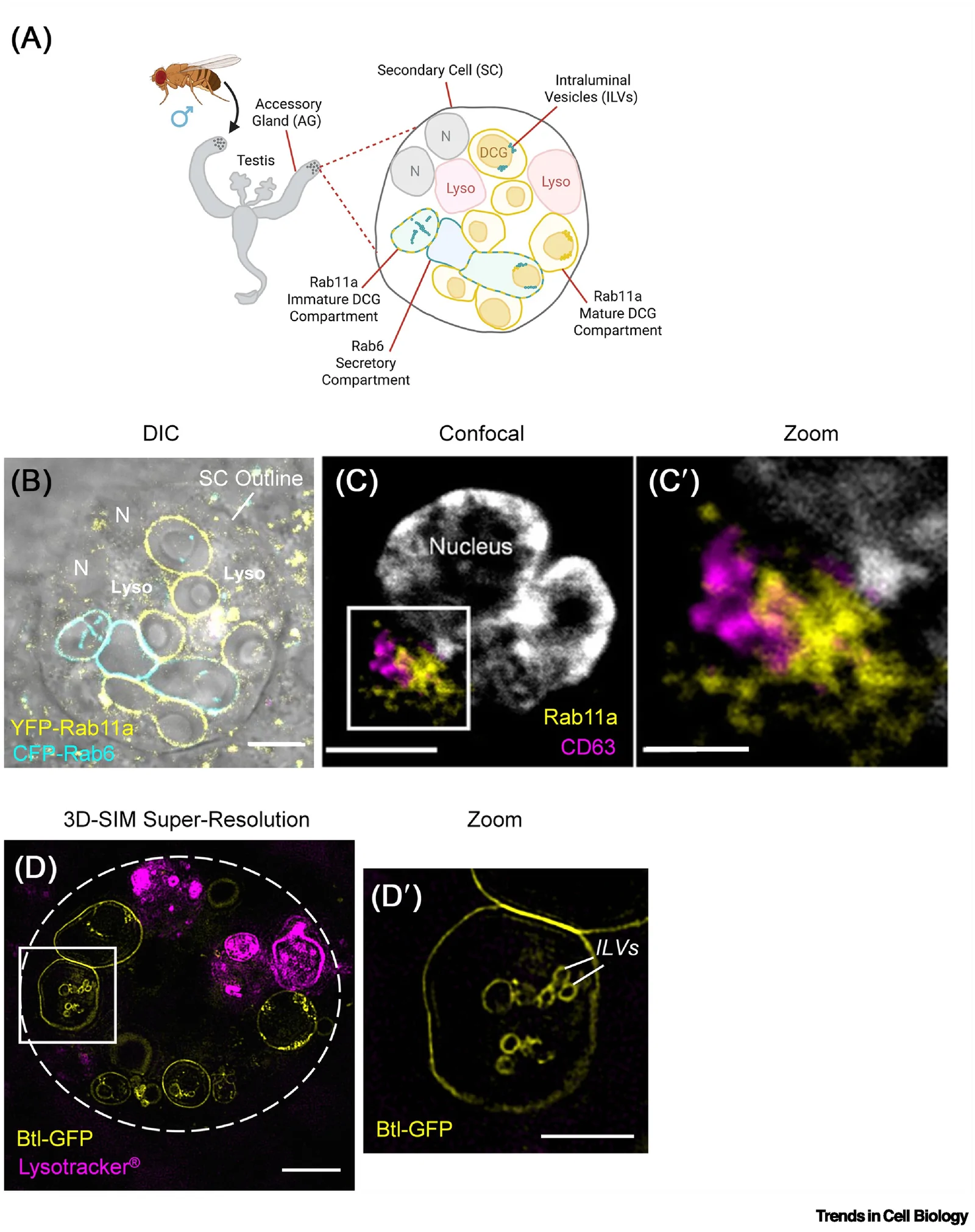
Exosome biogenesis in recycling endosomes of fly secondary cells. (A) Schematic of *Drosophila* secondary cell (SC) exosome biogenesis model in male bilobed accessory glands 16, 17. Binucleate (N) SCs are embedded within the glandular epithelial lining. SCs have lysosomes (Lyso) and Rab6-labelled Golgi-derived secretory compartments, which fuse with Rab11a-labelled endosomes, causing a gradual transition to Rab11a identity. Maturation of these Rab11a-labelled compartments is accompanied by Rab11a-exosome (ILV) biogenesis and protein aggregation events that generate a dense-core granule (DCG) [14]. The particularly large size of these nonacidic secretory endosomes facilitates visualisation of this Rab transition, exosome and DCG biogenesis 14, 18. (B) Single living SC with endogenous Rab6 and Rab11a proteins fluorescently labelled, showing secretory/DCG compartments (~5 μm diameter) and clustered ILVs contacting DCGs, schematically drawn in (A). (C,C’) As observed in many other mammalian cells, antibody-stained human HCT116 colorectal cancer cells have much smaller Rab11a compartments, prohibiting ILV resolution using standard light microscopy; late endosomes and lysosomes are labelled with CD63. (D,D’) Single living SC with the Rab11a-ILV/exosome transmembrane marker Breathless-GFP (Btl-GFP; *Drosophila* FGF receptor) overexpressed; lysosomes stained with LysoTracker Red®. Super-resolution 3D-SIM microscopy shows clustered ILVs (also see Zoom) contacting the endosomal limiting membrane; some ILVs are particularly large, but most are <100 nm in diameter [16]. Panel B is an image from [14, Figure 1]. Panels C and D are images from [16, Figures 3 and 1], respectively, of [16], with further labelling added. Scale bars are 5 μm or 2 μm (zooms). Panel A was created using BioRender (https://biorender.com/). All images were reproduced under the following Creative Commons licence: https://creativecommons.org/licenses/by/4.0/. CFP: Cyan Fluorescent Protein; FGF: Fibroblast Growth Factor; GFP: Green Fluorescent Protein; YFP: Yellow Fluorescent Protein; 3D-SIM: three-dimensional structural illumination microscopy.

Nonendosomal membranes can also contribute to MVE limiting membranes and ILVs, increasing exosome diversity; for example, multivesicular structures derived from viable mitochondria [**mitochondrion-derived vesicles (MDVs)**] fuse with MVEs, releasing ILVs formed from inner mitochondrial membranes, which are secreted as exosomes [13] (Figure 1B).

In addition, contrary to textbook models of regulated secretion, in which secretory and endosomal compartments remain separated, analysis in fly secondary cells demonstrated that Rab6-labelled, Golgi-derived secretory compartments mature via Rab11a-positive compartment inputs to assume recycling endosomal identity and produce Rab11a-exosomes (Figure 2 [14]). These vesicles carry membrane-associated proteins delivered through the regulated secretory pathway, for example, Rab6 [14] and glycosylphosphatidylinositol (GPI)-anchored proteins [22], in addition to recycling endosomal cargos. Rab11a-dependent maturation of Golgi-derived secretory compartments and ILV generation were subsequently demonstrated in human cells 23, 24, suggesting conservation from fly to human (Figure 1B).

**Autophagy** is traditionally viewed as a lysosome-dependent mechanism for organelle recycling, where ILV-containing **autophagosomes** fuse with lysosomes. In dying cells, such **autolysosome** generation can induce the secretion of immunogenic apoptotic exosome-like vesicles [11], which are distinct from plasma membrane-derived apoptotic bodies. Autophagosomes may also fuse with late endosomal MVEs, forming exosome-secreting **amphisomes**. Some of these exosomes are enriched in autophagy-related proteins [9] (Figure 1B). This autophagosome–endosome-dependent form of exosome release, termed **secretory autophagy**, also involves other endosomal membranes. Noncanonical Rab22A-associated autophagosome fusion with **early endosomes** (**rafeesomes**; Figure 1B) can generate exosomes carrying the innate immune activator STING [12]. Furthermore, amphisome fusion with lysosome-related secretory compartments in inflammatory mast cells permits the release of complex mixtures of amphisome-derived exosomes and soluble lysosome-derived immune mediators [10] (Figure 1B). Interestingly, the outer double membrane of autophagosomes is formed from Rab3a-labelled vesicles, which bud from recycling endosomal membranes at endoplasmic reticulum (ER) contact sites [8] (Figure 1A), suggesting that REs also contribute to autophagosome-dependent exosome formation.

For some exosome subtypes, specific mechanisms regulating exosome secretion have been defined (Figure 1B). Exosome secretion from amphisomes requires Rab27a, as well as many *Atg* genes that drive autophagosome formation [25]. Rab27a also regulates secretion from some secretory lysosomes 26, 27, while both Rab27a and Rab27b play roles in the secretion of late endosomal exosomes [28] and mitochondria-derived exosomes [29].

Although the membranes contributing to ILV generation are critical in determining the molecular profile of exosomes, this may not represent the last step in exosome biogenesis. Indeed, specific ILV subsets are not secreted but reassociate with endosomal limiting membranes via **retrofusion** [30]. Furthermore, the **corona** of ILVs, exosomes, and other EVs, consisting of proteins attached to the extravesicular membrane surface, is microenvironment-dependent and alters vesicular properties [31]. Exosomes may also be reloaded and resecreted when endocytosed, for example, generating exosomes carrying the ligand Wnt11a, which promotes breast cancer cell invasiveness [32].

In summary, contrary to previous views, intracellular compartment membranes contributing to exosome biogenesis are of diverse origin. Prior electron microscopic studies, which concluded that secretory MVEs are simply **late endosomes (LEs)**, may, therefore, require re-evaluation.

## Controlling exosome formation: compartmental membrane origin rules

Having wide-ranging exosome membrane origins inevitably affects the intracellular trafficking routes by which different exosome subtypes are released. A simple example is the blocking of late endosome fusion with lysosomes, which enhances exosome release [33]. Similarly, amphisome ILV secretion can be enhanced by fusion and fission with mast cell secretory compartments [10].

Signals that promote specific exocytosis events will also induce differential exosome subtype secretion. For example, activation of immunoglobulin E-mediated signalling stimulates mast cell exosome secretion [10], while increased extracellular matrix (ECM) stiffness promotes tumour exosome secretion, probably via the late endosomal pathway [34]. Reducing mechanistic target of rapamycin complex 1 (mTORC1) activity by nutrient depletion can favour recycling versus late endosomal trafficking, enhancing secretion of Rab11a-exosomes [16].

At least three general ILV biogenesis mechanisms have been reported. One requires the core **endosomal complexes required for transport (ESCRT)** proteins [35], which are major players in membrane budding and scission. Another involves a protein complex including a subset of ESCRTs, the accessory protein ALIX and Syntenin, which recruits specific membrane-bound cargos, such as Syndecan [36]. The third promotes membrane bending via ceramide production by membrane-associated neutral sphingomyelinase [37] (Figure 3A).

**Figure 3:**
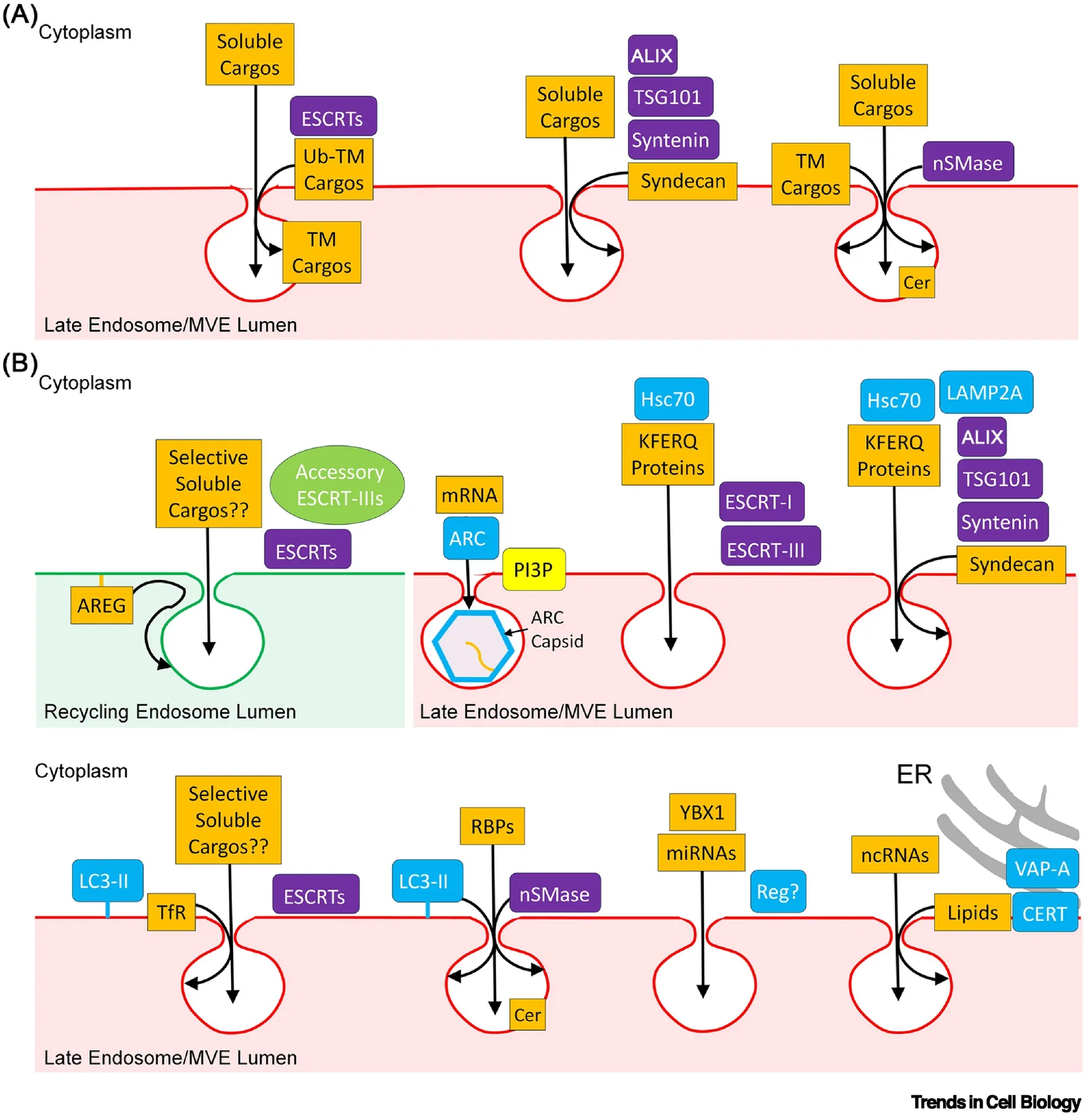
General and specialised mechanisms of intraluminal vesicle biogenesis and cargo loading within endosomes. (A) General mechanisms of intraluminal vesicle (ILV) biogenesis [1]. ILVs can be formed through sequential recruitment of endosomal sorting complex required for transport (ESCRT) proteins, with ESCRT-0 proteins playing key roles in sequestering transmembrane cargos with mono-/polyubiquitin (Ub) post-translational modifications. A second mechanism does not involve early ESCRT machinery but does include ESCRT-I protein TSG101 and ESCRT-III-associated protein ALIX, complexing with Syntenin and transmembrane cargo Syndecan, which promotes further selective cargo loading. A third mechanism recruits neutral sphingomyelinase (nSMase), generating ceramide (Cer), which induces membrane deformation. These late endosomal processes may occur in other MVEs. (B) Specialised ILV biogenesis mechanisms involving specific endosomes and/or specific cargo loading. In recycling endosomes, accessory ESCRT-III proteins selectively regulate ILV formation [19]. Membrane-associated amphiregulin (AREG) is a specific ILV cargo 16, 20. Most other mechanisms are associated with late endosomes but may be active in other MVEs. Specialised mechanisms include ARC capsid formation within ILVs, via a phosphatidyl-3-phosphate (PI3P)-dependent mechanism [38]. Complexes between Hsc70 and KFERQ-like domain-containing proteins can be loaded via LAMP2A-independent and LAMP2A-dependent mechanisms 39, 40. Lipid-conjugated LC3-II promotes ILV formation via an ESCRT-dependent mechanism, which loads specific transmembrane proteins, for example, transferrin receptor (TfR) [41], and a nSMase-dependent process, selectively loading RNA-binding proteins (RBPs) [42]. Condensates of specific miRNAs and YBX1 are packaged via an unknown mechanism into ILVs [43], while delivery of noncoding RNAs (ncRNAs) and lipids into ILVs is controlled by VAP-A and CERT at ER contact sites with MVEs. ALIX: ALG-2-interacting protein X; ARC: Activity Regulated Cytoskeleton-Associated Protein; CERT: ceramide transfer protein; ER: Endoplasmic Reticulum; KFERQ: Lysine (K) - Phenylalanine (F) - Glutamine (E) - Arginine (R) - Glutamine (Q); LAMP2A: Lysosome-Associated Membrane Protein 2A; MVE: multivesicular endosome; TM: Transmembrane; TSG101: Tumour Susceptibility Gene 101; VAP-A: Vesicle-Associated Membrane Protein-Associated Protein A.

Other regulators control subtype-specific exosome formation, sometimes collaborating with more general regulatory machinery. For example, accessory ESCRT-III proteins act with the core ESCRTs to selectively generate ILVs in Rab11a-labelled REs [19] (Figure 3B). Activity-regulated cytoskeleton-associated protein (ARC), an mRNA-binding protein with a retrovirus-like capsid-forming domain, interacts with phosphatidylinositol-3-phosphate, frequently associated with late endosomal trafficking, to form mRNA-containing ILVs [38] (Figure 3B). ILV loading of complexes containing the molecular chaperone Hsc70 and cytosolic proteins with a KFERQ-like peptide motif, implicated in endolysosomal targeting, can involve a lysosome-associated membrane protein 2A- (LAMP2A)-independent, ESCRT-dependent endosomal microautophagy mechanism in LEs [39], or a LAMP2A-dependent, ALIX–Syntenin-mediated ILV biogenesis mechanism in both early endosomes and LEs [40] (Figure 3B).

Furthermore, in addition to its central role in driving autophagy and amphisome formation, lipid-conjugated LC3 (ATG8 in yeast), LC3-II, can act independently of many other autophagy-associated *Atg* genes to generate late endosomal ILVs destined for secretion. Such mechanisms, known collectively as LC3-dependent EV loading and secretion (LDELS), can be ceramide-dependent [42] or ESCRT-dependent [41] (Figure 3B). Like Rab11a-exosome biogenesis, the link between LDELS and stress suggests a physiological role in intercellular homeostatic signalling; indeed, recently LDELS has been implicated in signalling by the nutrient-sensitive hormone leptin [44].

In summary, identifying compartmental trafficking pathways and specialised ILV biogenesis mechanisms for specific exosome subtypes provides opportunities to block these exosomes, characterise exosome subtype-specific functions, and identify more selective biomarkers. It remains possible that less extensively studied recycling MVEs could employ similar specialised mechanisms to LEs to generate exosome subtypes. Since the resulting exosomes would be derived from different endosomal membranes in distinct luminal microenvironments, however, they would likely have subtype-specific functions.

## Compartment identity affects exosome cargos and functions

ILVs are thought to assemble at microdomains of clustered lipids and proteins on the endosomal limiting membrane, which affect exosome cargos and functions [1]. For example, the tetraspanin CD63, which preferentially associates with LEs and, consequently, their ILVs, promotes cholesterol loading [45], driving intercellular cholesterol transfer. Recycling and late endosomal compartments traffic inherently different membrane-associated proteins, hence influencing ILV cargos. For example, the protumourigenic epidermal growth factor receptor (EGFR) ligand amphiregulin (AREG) selectively traffics to recycling endosomal Rab11a-exosomes in colorectal cancer cells (Figure 3B 16, 20).

The functional importance of membrane-specific cargos extends to nonendosomal membranes contributing to exosomes. For example, STING, an ER-derived immune-activating protein incorporated into autophagosomes and their ILVs (Figure 1B), is subsequently released from amphisomes and induces interferon secretion by target cells, promoting antitumour responses [12]. Similarly, fusion of MDVs with LEs produces ILVs enriched in mitochondrial inner membrane proteins, but the resulting exosome function is not defined [13]. While other compartment-specific cargos are donated by fusion of nonendosomal compartments with endosomes, for example, membrane-bound Rab6 from immature Golgi-derived secretory compartments loads onto Rab11a-exosomes [14], their functions in exosomes remain unclear.

Mechanisms involved in compartment- and subtype-specific exosome biogenesis can also determine cargo loading. For example, LAMP2A regulates ILV loading in early endosomes and LEs, preferentially selecting proteins with a KFERQ-like amino acid motif, such as HIF1-α, the master regulator of the hypoxic response [40] (Figure 3B). LDELS loads specific cytosolic cargos into ILVs, such as RNA-binding proteins [42], and membrane proteins, such as the transferrin receptor [41] (Figure 3B).

In addition, ER and MVE contact sites regulate the delivery of noncoding RNAs and lipids, such as ceramide and cholesterol, to ILVs controlled by the dynamic membrane tether VAP-A and ceramide transfer protein (CERT) [18] (Figure 3B). Other RNA-binding proteins and their RNAs incorporate into ILVs via alternative mechanisms. When complexed with Y-box binding protein 1 (YBX1) , specific miRNAs form phase-separated condensates, which are packaged into ILVs inside MVEs [43] (Figure 3B). Several cytosolic protein and RNA loading processes may be employed in recycling MVEs, in addition to late MVEs. The resulting exosomes, however, are likely to carry different surface proteins and, therefore, display different targeting and signalling properties, even if their cytosolic cargos are similar.

In summary, the diverse membrane origins contributing to ILV biogenesis generate multiple exosome subtypes, many with highly specialised cargos and functions. An important consequence is that any specific activity associated with a bulk EV preparation, even after ectosome removal, may be mediated by only a fraction of the vesicles.

## Microenvironmental impacts on intraluminal vesicle formation and function

Proteins associated with ILV membranes influence exosome distribution, targeting, and functions (Figure 4A). For example, the transmembrane protein Tetherin carries a C-terminal GPI anchor, which can insert into membranes. Tetherin-containing cancer cell exosomes carry the transmembrane protease MT1-matrix metalloprotease and remain attached to the plasma membrane when released [46], facilitating ECM degradation and promoting cellular invasiveness (Figure 4B).

**Figure 4:**
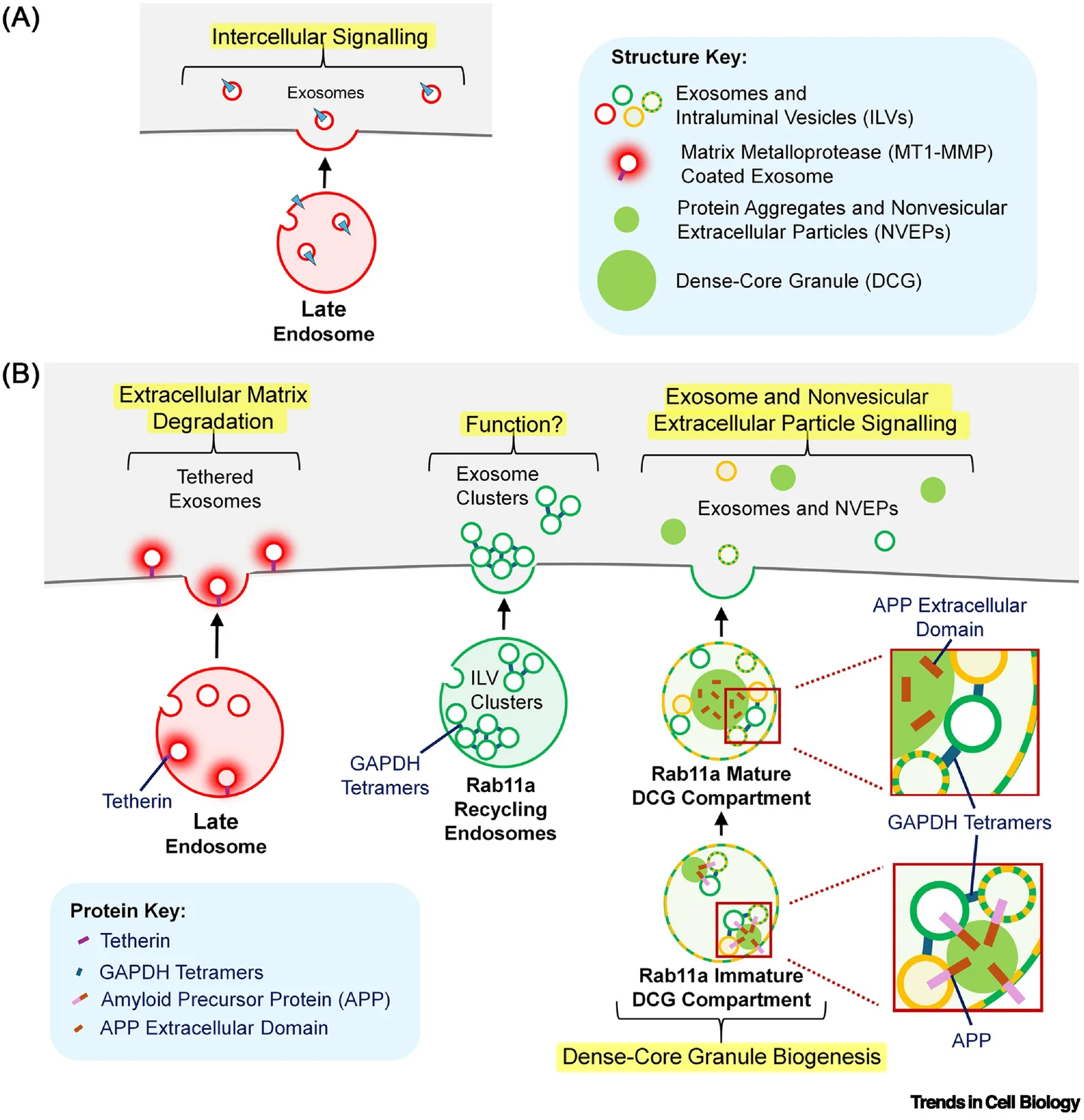
Microenvironmental interactions affect intraluminal vesicle and exosome function. (A) All secreted intraluminal vesicles (ILVs) secreted as exosomes carry membrane-associated cargos at their surface that can influence their targeting or signalling function. (B) Specific transmembrane proteins and other proteins that interact with the ILV and/or exosome surface can change vesicle properties and functions. Plasma membrane attachment by Tetherin’s GPI anchor enables exosome-associated MT1-matrix metalloprotease (MT1-MMP) to digest ECM [46]. Extravesicular membrane-associated glyceraldehyde-3-phosphate dehydrogenase (GAPDH) promotes clustering of ILVs and exosomes [47]. The surface-exposed transmembrane ILV protein amyloid precursor protein (APP) is involved in the formation of dense-core granules (DCGs), a physiological protein aggregation event associated with regulated secretory compartment maturation. This requires APP cleavage and leads to intracompartmental packaging of its extracellular domain and other signalling molecules into DCGs and degradation of the intracellular domain [48] (enlarged regions in boxes). These aggregation events may also lead to the generation of nonvesicular extracellular particles (NVEPs) with enhanced signalling activity, such as supermeres [49]. It is unclear whether GAPDH changes the functions of exosomes and other EVs outside cells, but it is required for normal extravesicular protein aggregation events within secretory DCG compartments. ECM: extracellular matrix; EV: extracellular vesicle; GPI: glycosylphosphatidylinositol.

In addition, early REs and LEs contain different soluble luminal macromolecules, made more diverse through nonendosomal inputs. The resulting microenvironments dynamically influence the ILV corona and, along with membrane-associated proteins, may modify exosome properties. Other molecules can contribute extracellularly to the exosome corona, further affecting function.

For example, the cytosolic glycolytic enzyme glyceraldehyde-3-phosphate dehydrogenase (GAPDH) is transported into the lumen of endosomal compartments via unconventional secretory mechanisms [50]. GAPDH associates with lipids, such as phosphatidylserine, on the outside of ILV and exosome/EV membranes. It promotes ILV clustering via its tetramerisation domain inside *Drosophila* secondary cell MVEs, altering ILV function [47] (see in the subsequent sections). The addition of GAPDH to human cell sEV preparations also promotes EV clustering, suggesting conservation of function [47] (Figure 4B).

Increasing evidence suggests that aggregating proteins interact with ILVs and exosomes to modulate their functions. ILVs inside melanosomes also act as apolipoprotein E (ApoE)-dependent primers for the amyloidogenic aggregation of Premelanosome protein (PMEL) cleavage fragments, which form a scaffold for melanin polymerisation [51]. *Drosophila* studies suggest that exosome-forming ILVs and the limiting membrane of recycling endosomal secretory compartments are involved in physiological protein aggregation prior to secretion. The aggregated proteins form a central dense-core granule (DCG), a characteristic feature of regulated secretory compartments [48] (Figure 4B).

Intriguingly, DCG formation is normally regulated by transmembrane amyloid precursor protein (APP), and the extracellular/luminal domain of APP is then cleaved and retained in the DCG, separating it from priming membranes (Figure 4B). Membrane–aggregate dissociation is disrupted by the expression of β-amyloid, the APP cleavage product that ultimately forms extracellular amyloid plaques in Alzheimer’s disease (AD), inducing endolysosomal trafficking defects that mirror early neuronal changes associated with degeneration [52]. Exosome-associated β-amyloid oligomers secreted from cells [53] may transfer toxic aggregates to other cells 54, 55. In fact, recent cryo-electron tomography studies of brains from patients with AD suggest that β-amyloid plaques contain structures resembling complexes between PMEL, ApoE, and ILVs in melanosomes [56], consistent with these structures playing an organising role in amyloid formation.

Cells also release **nonvesicular extracellular particles (NVEPs)**, protein–RNA aggregates with enhanced signalling activity, such as **supermeres**, which are secreted at high levels by cancer cells [49]. Their subcellular origin, however, remains unclear. Proteomic analysis of supermeres has identified the ECM protein TGFβ-induced (TGFBI) as the most abundant component, in addition to the APP extracellular domain, GPI-anchored proteins, and secreted proteases [49]. These proteins are also important components of *Drosophila* secondary cell DCGs, with TGFBI essential for DCG protein aggregation 22, 48, suggesting a potential link to supermere biogenesis.

In conclusion, the functions of specific ILVs and exosomes are not only influenced by the signals that they carry but also by other transmembrane and surface proteins, which alter their interactions. The extravesicular microenvironment plays a key role in these mechanisms, emphasising the critical importance of understanding the origins of different ILVs and exosomes in determining their physiological and pathological functions.

## Unique functions for specific exosome subtypes in disease

Although the challenges of separating exosomes from other sEVs often make it difficult to definitively assign exosome-specific functions [15], these vesicles are thought to play important roles in both physiology and major human diseases 2, 6. For example, extensive cancer studies suggest that exosomes and other sEVs contribute to each of the ‘hallmarks of cancer’ [57], regulating metastasis, exerting immunological effects, transferring tumorigenic properties, and promoting treatment resistance [58]. Exosomes are not only vehicles for signals but can also increase signal potency. Of note, exosome membrane-associated AREG can outcompete a therapeutic anti-EGFR antibody at much lower concentrations than soluble AREG 16, 20.

Several exosome subtype-specific mechanisms reviewed here play important roles in cancer, for example, 12, 16, 40 (Figures 3B and 4B), often because they are induced by microenvironmental stresses, such as hypoxia or nutrient stress, both key factors in tumour adaptation. Exosomes and other sEVs are also potential intercellular communicators of stress-associated homeostatic imbalance in AD [54], gestational diabetes [59], and other endocrinological diseases [60]. Determining additional roles of specific exosome subtypes in pathological stress-induced signalling is, therefore, likely to be important in developing therapies and diagnostics.

Indeed, as interest in clinical applications of exosomes has widened, so has the need for rapid, high-throughput assays. To assay sEVs in the clinic, improved separation, detection, and protein/miRNA/mRNA quantification are needed. Separation methods have expanded to include electrochemistry, imaging, and microfluidics [61]. For exosome subtype-specific detection, a surface immunoaffinity step is likely to be critical, although identification of exosome subtype-specific lipids or physical properties might lead to alternative strategies. However, for some exosome subtype-specific biogenesis mechanisms discussed earlier, which are defined by the loading of cytosolic proteins (e.g., 38, 43), an immunoaffinity approach is currently not feasible.

In cases where proteins associated with exosome membranes have established pathological effects, combining approaches to suppress vesicle activity and reduce biogenesis may be essential. For example, antibodies targeting tumour exosome-associated immune checkpoint ligand PD-L1, which suppresses the systemic immune response to cancer cells, have shown some efficacy in animal models 62, 63. While reducing the production of these exosomes would complement this approach, identifying strategies for subtype-specific exosome blockade is critical to avoid major side effects [64].

Finally, a key challenge in developing RNA-based and other therapies is selective delivery and uptake. Many approaches are being developed to produce engineered exosomes with targeting specificity to deliver cargos, which also include chemotherapeutics and agents that can be integrated with, for example, sonodynamic, photothermal, and photodynamic therapies 65, 66, 67. In these and other clinical applications, the mechanisms by which the therapeutically active vesicles are generated are largely unexplored, even though this could drastically influence their targeting and signalling activity.

## Concluding remarks and future perspectives

Rather than thinking of exosomes as one class of EVs, recent studies have highlighted their heterogeneous nature and the contributions of different compartments and membranes to exosome subtypes with specialised activities. These discoveries emphasise the importance of identifying and better characterising exosome subtype-specific mechanisms in health and disease, as a powerful alternative to screening for subtype-specific markers in heterogeneous sEV preparations, where the vesicular origin and functional significance may be unclear.

Understanding the basis of exosome subtype generation also suggests new ways to investigate sEVs that might reveal further membrane origins and mergers. For example, **Rab** proteins are low-abundance cargos in sEVs, but they can signpost the membrane origin of exosomes 14, 16, potentially highlighting trafficking mechanisms that mediate their biogenesis. In addition, further consideration of the link between protein aggregates and ILVs/exosomes may improve our interpretation of physiological exosome signalling, while also explaining how this malfunctions in disease. We envisage that a greater focus on subtype-specific exosome assembly mechanisms (see Outstanding questions) will facilitate the identification of exosomes with key signalling activities, thereby providing a robust rationale to develop approaches that selectively detect and block them in disease, or exploit them in biodelivery.

Outstanding questions In addition to the organelles discussed, do other organelle membranes contribute to intraluminal vesicle and exosome formation?

Are the **Rab GTPase** cargos, which are found in many small extracellular vesicle proteomic studies, signatures of the origin of additional secretory and endolysosomal membranes that contribute to exosomes, including alternative recycling endosomal pathways?

Do the different membrane origins of specific exosome subtypes affect the recipient cell's exosome uptake pathway, and how cargos reach their subcellular destinations?

Which exosome subtype-specific biogenesis mechanisms operate in both late and recycling endosomes?

How do the specialised intraluminal vesicle-generating lipid microdomains on endosomal limiting membranes differ between mechanisms controlling the formation of specific exosome subtypes?

In a similar fashion to ubiquitinated proteins being specifically targeted and loaded onto late endosomal intraluminal vesicles, is there an alternative post-translational modification for recycling endosomal intraluminal vesicles?

What other signalling pathways control the different intraluminal vesicle-generating mechanisms employed by cells and determine whether these intraluminal vesicles are secreted?

Can membrane-associated surface proteins that label specific exosome subtypes be identified, and can these be used to diagnose different diseases using single-vesicle analysis?

Is the link between intraluminal vesicles and protein aggregation important in other amyloidogenic diseases, and does it play a role in physiological exosome signalling?

Do the intraluminal vesicle precursors of Rab11a-exosomes have a specialised role in protein aggregation, or do other intraluminal vesicles perform similar roles in the appropriate microenvironment?

What connections exist between intraluminal vesicle-associated protein aggregates and nonvesicular extracellular particles, such as supermeres?

Alt-text: Outstanding questions

## Glossary

**Amphisomes**: hybrid compartments produced by LE and LC3-II-positive autophagosome fusion; they can contain endosomal ILVs and LC3-II-positive ILVs formed from autophagosome-derived membrane.
**Autolysosomes**: compartments formed by an autophagosome or an amphisome fusing with a lysosome.
**Autophagosomes**: double-membrane-bound compartments formed during autophagy.
**Autophagy**: a mechanism for delivering cytoplasmic material to lysosomes for degradation, also involved in specialised exosome biogenesis and secretion.
**Corona**: protein and other macromolecular coatings on the external surface of exosomes and other EVs.
**Early endosomes**: compartments formed from vesicles invaginating from the plasma membrane; their contents are actively distributed between LEs and REs.
**Ectosomes**: EVs with diverse functional activities, formed from the outward budding of the plasma membrane.
**Endosomal complexes required for transport** (**ESCRT**): a group of cytosolic proteins with functions including regulating the formation of ILVs and certain types of ectosomes.
**Endosomes**: compartments mostly derived from plasma membrane endocytosis.
**Exosomes**: formally defined as sEVs, formed as ILVs in compartments with endosomal origin and subsequently secreted upon fusion of the resulting MVEs with the plasma membrane.
**Extracellular vesicles** (**EVs**): membrane-delimited particles, secreted by cells, which can deliver bioactive molecules to recipient cells and cannot self-replicate.
**Intraluminal vesicles** (**ILVs**): formed from the inward budding of the limiting membrane of an MVE or another type of MVB.
**Late endosomes (LEs)**: compartments formed from early endosomal membranes, involved in macromolecular degradation and exosome biogenesis.
**Mitochondrion-derived vesicles** (**MDVs**): derived from the outward budding of the inner mitochondrial membrane. MDVs fuse with LEs, forming exosomes that can transfer mitochondrial constituents between cells.
**Multivesicular bodies** (**MVBs**): compartments containing ILVs, a subset of which are MVEs.
**Multivesicular endosomes** (**MVEs**): a type of endosomal compartment in which the limiting membrane has invaginated to form ILVs, which can be released as exosomes upon MVE fusion with the plasma membrane.
**Nuclear envelope-derived MVBs** (**NE-MVBs**): MVBs formed from the outward budding of the inner nuclear envelope into the cytosol and the formation of ILVs; subsequently released as nonendosomal ‘exosomes’ when NE-MVBs fuse with the plasma membrane.
**Nonvesicular extracellular particles (NVEPs)**: particles secreted by cells that are not enclosed by lipid membranes.
**Rab GTPases**: a large family of small monomeric G proteins that act as molecular switches, controlling intracellular membrane trafficking, for example, Rab6 (Golgi), Rab7 (LEs), and Rab11a (specific slow REs; Rab11 in *Drosophila*).
**Rab11a-exosomes**: exosomes made in Rab11a-labelled REs.
**Rafeesomes**: formed by Rab22a-mediated fusion of noncanonical autophagosomes with early endosomes. They contain ILVs, loaded with STING, which are secreted as specialised exosomes.
**Recycling endosomes** (**RE**s): compartments formed from early endosome tubulation, sort and return molecules to the cell surface for reuse, and are also involved in exosome biogenesis.
**Retrofusion**: back-fusion of ILVs to the endosomal limiting membrane.
**autophagy**: a cellular release mechanism involving autophagosomes and endolysosomes.
**Small EVs** (**sEVs**): 30–150 nm diameter EVs, including exosomes and some ectosomes.
**Supermeres**: extracellular NVEPs with signalling activities, formed from proteins, nucleic acids, and lipids.
